# Multi-omics analysis reveals shared diagnostic and therapeutic targets in endometriosis and recurrent implantation failure

**DOI:** 10.1038/s41598-025-28877-8

**Published:** 2025-12-29

**Authors:** Jie YU, Wei WANG, Qiong LI, Lan LAN, Li-Li JIANG, Xin-Rong HE, Xiao-Wei JIANG, Yu-lin YAN, Xiao-Ming YAO, Meng-Yue WANG, Ping-Mei DUAN, Lin-Chun HUANG, Hai-Feng QI, Ting-He YU

**Affiliations:** 1 Department of Obstetrics and Gynecology, The Second Affiliated Hospital of Chongqing Medical University, Chongqing, China; 2 Women and Children’s Hospital of Chongqing Medical University (Chongqing Health Center for Women and Children), Chongqing, China

**Keywords:** Endometrial receptivity, Endometriosis, Recurrent implantation failure, Weighted gene co-expression network analysis, Core genes, Diagnostic biomarkers, Protein-protein interaction networks, Immunochemistry

## Abstract

Endometrial receptivity is essential for successful pregnancy, and endometriosis is widely recognized as a disruptor of this process. Poor endometrial receptivity is also a key factor contributing to recurrent implantation failure. Although some molecular mechanisms related to endometrial receptivity have been identified, their specific roles in endometriosis and recurrent implantation failure remain unclear. This study aimed to elucidate the shared molecular mechanisms affecting endometrial receptivity in endometriosis and recurrent implantation failure using multi-omics data analysis. We sourced datasets from the NCBI GEO database and employed weighted gene co-expression network analysis to identify gene modules associated with these conditions, followed by gene ontology (GO) and Kyoto Encyclopedia of Genes and Genomes (KEGG) pathway analyses. Single-cell sequencing analysis and immunofluorescence were used for expression analysis. We identified 3690 and 4892 upregulated genes and 2675 and 5065 downregulated genes in endometriosis and recurrent implantation failure, respectively. Functional enrichment analysis and validation identified 15 hub genes including SRPRB, SLC35B1, and SLC25A6. Receiver operating characteristic curve analysis demonstrated that these genes are associated with high diagnostic accuracy. Single-cell sequencing analysis indicated that these genes are predominantly expressed in basal epithelial cells, with RBM3 being particularly prominent. This study provides new insights into the molecular mechanisms underlying endometrial receptivity and identifies potential targets for the diagnosis and treatment of endometriosis and recurrent implantation failure.

**Supplementary Information:**

The online version contains supplementary material available at 10.1038/s41598-025-28877-8.

## Introduction

 Endometrial receptivity plays a fundamental role in embryo implantation and placental development. Therefore, it represents a pivotal therapeutic focus in reproductive medicine^1,2^. Clinically, recurrent implantation failure (RIF)—defined as the absence of clinical pregnancy after ≥ 3 transfers of high-quality embryos—remains a persistent challenge in assisted reproductive technology^3^. This condition predominantly arises from compromised endometrial-embryo crosstalk. Multifaceted receptivity dysfunction accounts for over half of RIF cases. Specifically, dysregulated inflammatory microenvironments have elevated pro-inflammatory cytokines and altered uNK/Treg cell dynamics. These changes create embryotoxic conditions that disrupt implantation signaling. Concurrently, structural and molecular perturbations—including impaired glandular development, aberrant vascular remodeling, and diminished adhesion molecule expression (e.g., integrin αvβ3 and L-selectin ligands)—hinder blastocyst attachment and invasion. Hormonal desynchronization resulting from progesterone resistance further exacerbates this failure by impairing the closure of the implantation window, while dysfunctional PI3K/AKT and Wnt/β-catenin pathways cripple decidual transformation and embryo-endometrial communication^4–7^.

Endometriosis (EM), prevalent in 20–50% of infertile women^8^, synergistically amplifies these defects. Local inflammatory processes driven by IL-1β and TNF-α overexpression propagate cytotoxic niches, Ectopic lesions cause direct physical damage to implantation sites. Additionally, shared molecular abnormalities—such as mitochondrial oxidative stress, hyperactivated PI3K/AKT signaling that impairs trophoblast invasion, and aberrant estrogen receptor activity—collectively destabilize endometrial function^9–13^. Importantly, hidden (occult) EM underlies approximately one-third of idiopathic RIF cases, demonstrating an etiological continuum between these conditions that demands comparative mechanistic exploration^14,15^.

Conventional single-omics methodologies, while valuable, fragment this biological complexity by isolating molecular strata from their cellular and microenvironmental context Multi-omics integration directly addresses this limitation by cross-validating molecular networks through complementary approaches, resolving spatially heterogeneous cellular ecosystems, and pinpointing clinically translatable biomarkers with enhanced robustness. Building on this paradigm, our study integrates transcriptomic co-expression networks, immune landscape deconvolution, and single-cell spatial mapping to construct an analytical framework capable of uncovering previously unrecognized regulatory circuits. This methodological synergy facilitates the identification of therapeutically relevant targets and the development of clinical prediction models for early intervention in high-risk populations.

To address the unmet need for systematic comparisons of EM-RIF (Endometrial Receptivity Implantation Failure), we identify key hub genes that converge mechanistically to govern receptivity failure. We then validate the diagnostic utility of these genes across omics platforms and delineate pathogenic immune-metabolic networks that reveal novel targets for precision therapy.

## Materials and methods

### Data acquisition and preprocessing

The raw expression profile datasets GSE25628 and GSE51981 for endometriosis and its normal controls were downloaded from the NCBI GEO database^16^ with GSE25628 serving as the training set and GSE51981 as the validation set. Additionally, microarray datasets GSE111974 and GSE26787 for RIF were obtained, using GSE111974 as the training set and GSE26787 as the validation set. The downloaded data were processed, standardized, and log2-transformed probe expression matrices, along with corresponding platform annotation files. Probes were matched one-to-one with gene symbols, and probes without a gene symbol match were removed. For probes mapping to the same gene, the average expression value of these probes was taken as the final expression value for that gene. Furthermore, a single-cell RNA-sequencing (scRNA-seq) dataset GSE216748^17^ of the endometrial organoids of Asherman’s syndrome (AS) was downloaded from the GEO database, which included three AS pre-treatment samples and three controls, to determine the expression levels of target genes in cells. Table 1 summarizes the detailed information of these selected datasets.

**Table 1:** Information of the GEO datasets enrolled in study.

Diseases	Dataset usage	Accession ID	Sample type	Sequencing type	Diseases samples	Normal samples	Platform
Endometriosis	Training set	GSE25628	endometrium	RNA-seq	16	6	GPL571[HG-U133A_2] Affymetrix Human Genome U133A 2.0 Array
	Validation set	GSE51981	endometrial tissue	RNA-seq	77	71	GPL570[HG-U133_Plus_2] Affymetrix Human Genome U133 Plus 2.0 Array
Recurrent implantation failure	Training set	GSE111974	endometrial tissue	RNA-seq	24	24	GPL17077Agilent-039494 SurePrint G3 Human GE v2 8 × 60 K Microarray 039381 (Probe Name version)
	Validation set	GSE26787	endometrium	RNA-seq	5	5	GPL570[HG-U133_Plus_2] Affymetrix Human Genome U133 Plus 2.0 Array
Endometrial organoids of Asherman’s syndrome	Single-cell data analysis	GSE216748	endometrium	single cell RNA-seq	3	3	GPL24676Illumina NovaSeq 6000 (Homo sapiens)

### Differentially expressed genes (DEGs) analysis

The classic Bayesian method provided by the R package “limma”^18^ (version 3.10.3) was utilized to perform differential expression analysis between disease samples and normal samples in the training sets of endometriosis and RIF. After analysis, all genes were assigned corresponding p.value values. Genes with a differential expression threshold of p.value < 0.05 were selected as DEGs. Volcano plots were created to visualize the expression patterns of DEGs.

### Screening of disease-related module genes by weighted gene co-expression network analysis (WGCNA)

Based on the expression levels of DEGs in the training sets of endometriosis and RIF, the R package “WGCNA”^19^ (version 1.61) was used to analyze the input genes and identify gene set modules that are highly co-expressed with endometriosis and RIF, respectively. First, a series of power values were set, and the squared correlation coefficient between connectivity k and p(k), as well as the mean connectivity, were calculated for each power value. An appropriate soft power value was selected to ensure that the connections between genes in the network followed a scale-free network distribution. Second, using clustering and dynamic tree cutting methods with parameters minModuleSize = 30 and mergeCutHeight = 0.25, highly correlated genes were aggregated into modules. Finally, with sample groups (diseases and normal controls) as phenotypes, the correlation between modules and phenotypes was calculated. Modules highly correlated with sample groups and with *p* < 0.05 were selected as disease-related key modules. The genes in these modules were identified as endometriosis-related module genes and RIF-related module genes, respectively.

### Identification and enrichment analysis of shared susceptibility genes from endometriosis and RIF

Based on the analysis results of WGCNA, the intersection of upregulated genes and the intersection of downregulated genes between endometriosis-related module genes and RIF-related module genes were taken. These two sets of intersecting genes were combined to obtain shared susceptibility genes. Subsequently, to explore the biological functions and molecular mechanisms of these genes, the R package “clusterProfiler”^20^(version 4.14.4) was used to perform Gene Ontology (GO)^21^ and Kyoto Encyclopedia of Genes and Genomics (KEGG)^22,23^ pathway enrichment analyses. The GO enrichment analysis covered three aspects: biological processes (BPs), cellular components (CCs), and molecular functions (MFs). Results with *p* < 0.05 were selected as significantly enriched.

### Development of diagnostic genes associated with endometriosis and RIF

The R package “limma” was used to validate the differential expression of shared susceptibility genes in the training and validation sets of endometriosis and RIF. Genes with significant test results (*p* < 0.05) and consistent up/down-regulation trends were selected as hub genes. Subsequently, the R package “pROC”^24^ (version 1.12.1) was utilized to draw receiver operating characteristic (ROC) curves for these genes and calculate the area under the curve (AUC) values to assess their diagnostic accuracy. Genes with AUC > 0.65 in both the training and validation sets were retained as diagnostic genes for endometriosis and RIF.

### Evaluation of immune cell infiltration

To evaluate the immune microenvironment characteristics of endometriosis and RIF, the R package “CIBERSORT”^25^ method was used to determine the infiltration proportions of 22 immune cell types in disease samples and normal control samples of the endometriosis training set GSE25628 and the RIF training set GSE111974. Subsequently, Spearman correlation analysis was employed to explore the correlations between diagnostic genes and different cell types, with the results displayed in a heatmap.

### Expression analysis of diagnostic genes in single-cell data

The scRNA-seq dataset GSE216748 was downloaded, and the R package “Seurat” (version 4.1.1) was used for single-cell feature research to determine the expression levels of diagnostic genes in cells. First, genes expressed in fewer than three cells in the samples and cells expressing fewer than 200 genes were excluded. Then, quality control of cells was performed based on the number of genes expressed in the count matrix and the percentage of mitochondrial gene counts, filtering out cells with more than 6,000 genes and cells with mitochondrial gene counts exceeding 20%. Subsequently, the library size normalization method was applied, and the data matrix was normalized using the “scanpy.pp.normalize_total” function from the Python package “Scanpy” to obtain a log-normalized data matrix for subsequent analysis^26^. Dimensionality reduction and unsupervised clustering were performed based on the workflow in the Python package “Scanpy”. The “scanpy.pp.highly_variable_genes” function was used to select the top 4,000 highly variable genes for downstream analysis. The impact of the total count and the percentage of mitochondrial genes expressed in each cell was regressed out using the “scanpy.pp.regress_out” function. Additionally, each gene was scaled to unit variance using the “scanpy.pp.scale” function (with the parameter “max_value = 10”). After data preprocessing, principal component analysis (PCA) was conducted to reduce the dimensionality of the data. The dimensionality of the dataset was further reduced using UMAP implemented by the “scanpy.tl.umap” function. Leiden clustering was applied to cluster cells in the neighborhood graph^27^.

### Patients and sample collection

This study protocol was approved by the Ethics Committees of Chongqing Medical University and Chongqing Maternal and Child Health Care Hospital (approval no. 2021-006). All experiments and practices adhered to medical ethical principles and the Declaration of Helsinki. Written informed consent was obtained from all female patients before sample collection.

The endometrial clinical samples were collected from the Reproductive Medicine Center of Chongqing Maternal and Child Health Care Hospital from 2023 to 2024. The enrolled females included 9 endometriosis patients, 12 RIF patients, and 12 healthy controls matched for age and body mass index (BMI). The specific grouping information is as follows: Normal control group, females undergoing in vitro fertilization (IVF) treatment due to tubal factors or male factors, and achieving clinical pregnancy after the first embryo transfer. Endometriosis group, females undergoing IVF treatment for primary infertility, achieving clinical pregnancy after the first embryo transfer, and previously diagnosed with endometriosis. RIF group, females aged 25–39 with regular menstrual cycles, undergoing IVF treatment, and failing to achieve clinical pregnancy after transferring at least four high-quality embryos in at least three fresh or frozen IVF cycles.

All the enrolled women had regular menstrual cycles and had abstained from hormonal medications for a minimum of 6 months. Females with the following conditions were excluded: polycystic ovary syndrome, hydrosalpinx, intrauterine lesions (congenital uterine anomalies, uterine fibroids, endometrial polyps, and intrauterine adhesions), positive lupus anticoagulant or anti-cardiolipin antibodies, and chromosomal abnormalities, etc. The normal control group also excluded endometriosis. Detailed clinical and pathological information of the three groups is summarized in Supplementary Table S1.

During the proliferative phase, endometrial tissues were collected using a disposable endometrial sampler during hysteroscopy or endometrial biopsy. Immediately after sampling, the samples were placed in sterile containers, immersed in pre-chilled PBS, and promptly transported to the laboratory for further processing. A portion of the samples was washed with PBS and immediately frozen in liquid nitrogen or stored at −80 °C for later use. Another portion was fixed in 4% paraformaldehyde for 24 h and then embedded in paraffin for histological analysis.

### Immunofluorescent staining

The expression of diagnostic genes (SRPRB, RBM3, INSIG2, GYG1, and FBXW2) in different tissues was investigated to verify their differential expression. Endometrial tissue samples were collected from 12 normal controls, 9 endometriosis patients, and 12 RIF patients. Tissue sections were dewaxed using a xylene and ethanol gradient method. Subsequently, the sections were placed in pH 8.0 EDTA alkaline retrieval solution and microwaved for antigen retrieval. To prevent non-specific binding of immunoglobulins, the sections were blocked with 10% normal goat serum (Solarbio, SL038,China). Next, the following primary antibodies were added and incubated overnight at 4 °C: Vimentin (Abcam, ab8978, 1:100), SRPRB (Abcam, ab236725, 1:100), RBM3 (Abcam, ab236725, 1:100), INSIG2 (Abcam, ab289767, 1:100), GYG1 (Immunoway, YN7222, 1:100), and FBXW2 (Immunoway, YT7491, 1:100). Then, the sections were incubated with corresponding secondary antibodies(1:1000, Abcam, UK). Fluorescent signals were visualized using TYR-520 and TYR-570 fluorophores, and cell nuclei were counterstained with DAPI. Subsequently, the sections were mounted with an anti-fade mounting medium. For imaging, digital images were captured from six random areas of each slide using an optical microscope (BX51, Olympus, Japan) and ISCapture software (Tucsen, China). Finally, the intensity of immunostaining was quantitatively analyzed using Image Pro Plus software (version 6.0, Media Cybernetics, USA).

### Western blot analysis

Endometrial tissue samples were collected from 12 normal controls, 9 endometriosis patients, and 12 RIF patients for total protein extraction. The samples were lysed using RIPA lysis buffer (Beyotime, P0013E, China) supplemented with protease and phosphatase inhibitors to prevent protein degradation and loss of phosphorylation. The extracted proteins were separated by 10% SDS-PAGE and then transferred onto polyvinylidene difluoride (PVDF) membranes (Millipore). The membranes were blocked with a rapid blocking solution (NCM Biotech) at room temperature for 30 min to prevent non-specific binding. Subsequently, the membranes were incubated overnight at 4 °C with the following primary antibodies: anti-SRPRB (Abcam, ab236725), RBM3 (Abcam, ab236725), INSIG2 (Abcam, ab289767), GYG1 (Immunoway, YN7222), FBXW2 (Immunoway, YT7491), and GAPDH (Proteintech, 60004-1-IG). The next day, appropriate secondary antibodies were added: goat anti-mouse IgG (Proteintech, SA00001-1) and goat anti-rabbit IgG (Proteintech, SA00001-2), and the membranes were incubated at room temperature for 1 h. Protein signals were visualized using an enhanced chemiluminescence (ECL) detection kit (MCE; HY-K1005). The grayscale values were analyzed using ImageJ software (1.53T, National Institutes of Health (NIH), USA) to determine the relative levels of protein expression.

### Statistical analysis

Statistical analyses were performed using R software (version 4.2) and GraphPad Prism software (version 9). Experimental results are presented as mean ± standard deviation (SD), with data derived from at least three independent experiments. Intergroup comparisons were made using one-way analysis of variance (one-way ANOVA), followed by Tukey’s post hoc test. A p-value less than 0.05 was considered statistically significant. Levels of significance are indicated as follows: ns, no significance; *p < 0.05; **p < 0.01; ***p < 0.001; ****p < 0.0001.

## Results

### Screening of DEGs for endometriosis and RIF

**Fig. 1:**
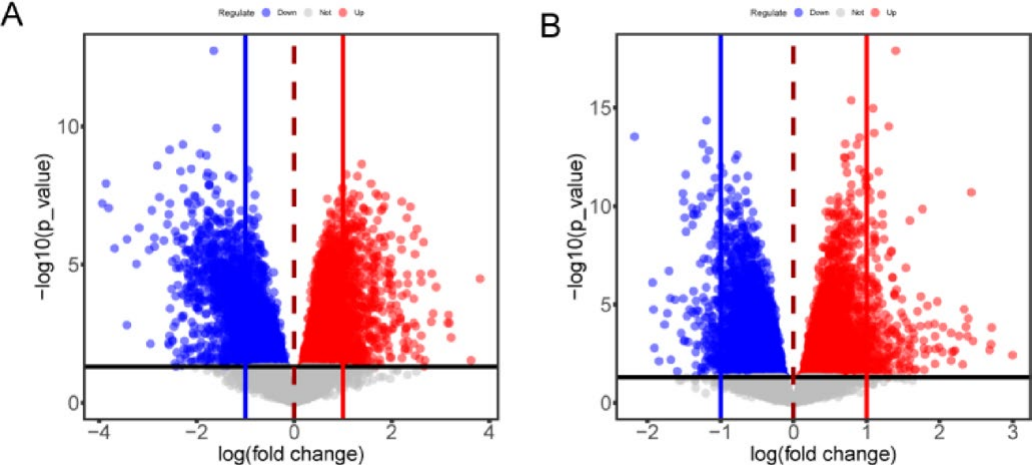
Volcano plots exhibit DEGs of endometriosis (**A**) and RIF (**B**). Red dots represent up-regulated genes, blue dots represented down-regulated genes, and grey dots represent non-significant genes.

In the endometriosis training set GSE25628, a total of 6,365 DEGs were detected between endometriosis and normal control samples, including 3,690 upregulated genes and 2,675 downregulated genes, as shown in the volcano plot (Fig. 1A). In the RIF training set GSE111974, a total of 9,957 DEGs were detected between RIF and normal control samples, including 4,892 upregulated genes and 5,065 downregulated genes, also presented in a volcano plot (Fig. 1B).

### Identification disease-related module genes in endometriosis and RIF

**Fig. 2:**
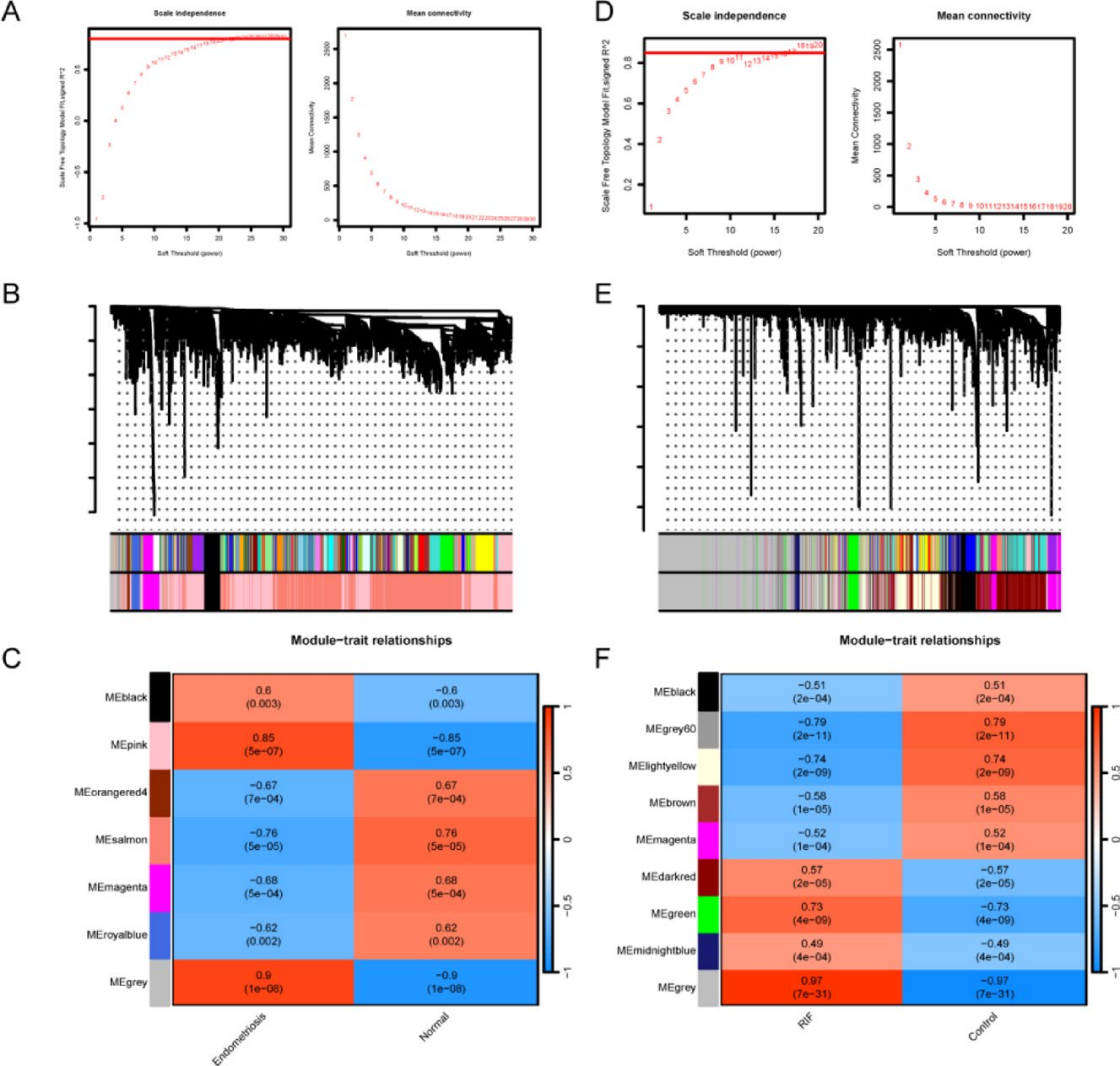
Identification of disease-related module genes via WGCNA in the GSE25628 dataset of endometriosis and the GSE111974 dataset of RIF. (**A**) The scale-free topology index and average connectivity for soft power threshold in endometriosis. (**B**) Cluster dendrogram of co-expressed modules in endometriosis. (**C**) Heatmap of module-trait correlations in endometriosis. (**D**) The scale-free topology index and average connectivity for soft power threshold in RIF. (**E**) Cluster dendrogram of co-expressed modules in RIF. (**F**) Heatmap of module-trait correlations in RIF.

WGCNA analysis was performed on the DEGs in GSE25628 dataset of endometriosis. Topological calculations were conducted with soft thresholds ranging from 1 to 30, determining the optimal soft threshold β = 24 (Fig. 2A). Based on clustering and dynamic tree cutting, highly correlated genes were grouped into modules, which were then clustered. Modules with a dissimilarity coefficient less than 0.25 were merged, and after excluding the MEgrey module, a total of 6 modules were obtained (Fig. 2B). Subsequently, the correlation between each module’s eigengene and the endometriosis phenotype was explored. The module-trait correlation heatmap showed that the MEplink module was most significantly correlated with endometriosis (|r| = 0.85 and *p* = 5e-07) (Fig. 2C). Therefore, this module was identified as the key module for endometriosis, containing 1,700 genes. Similarly, WGCNA analysis was also conducted on the DEGs of RIF in GSE111974. The optimal soft threshold β was selected as 15 (Fig. 2D). Based on clustering and dynamic tree cutting, highly correlated genes were grouped into modules, and modules with a correlation coefficient greater than 0.75 were merged. After excluding the MEgrey module, a total of 8 modules were integrated (Fig. 2E). The MElightyellow module was found to be significantly correlated with RIF (|r| = 0.74 and *p* = 2e-09) and was selected as the key module for further analysis, containing 1,600 genes (Fig. 2F).

### Shared susceptibility genes in endometriosis and RIF and enrichment analysis

**Fig. 3:**
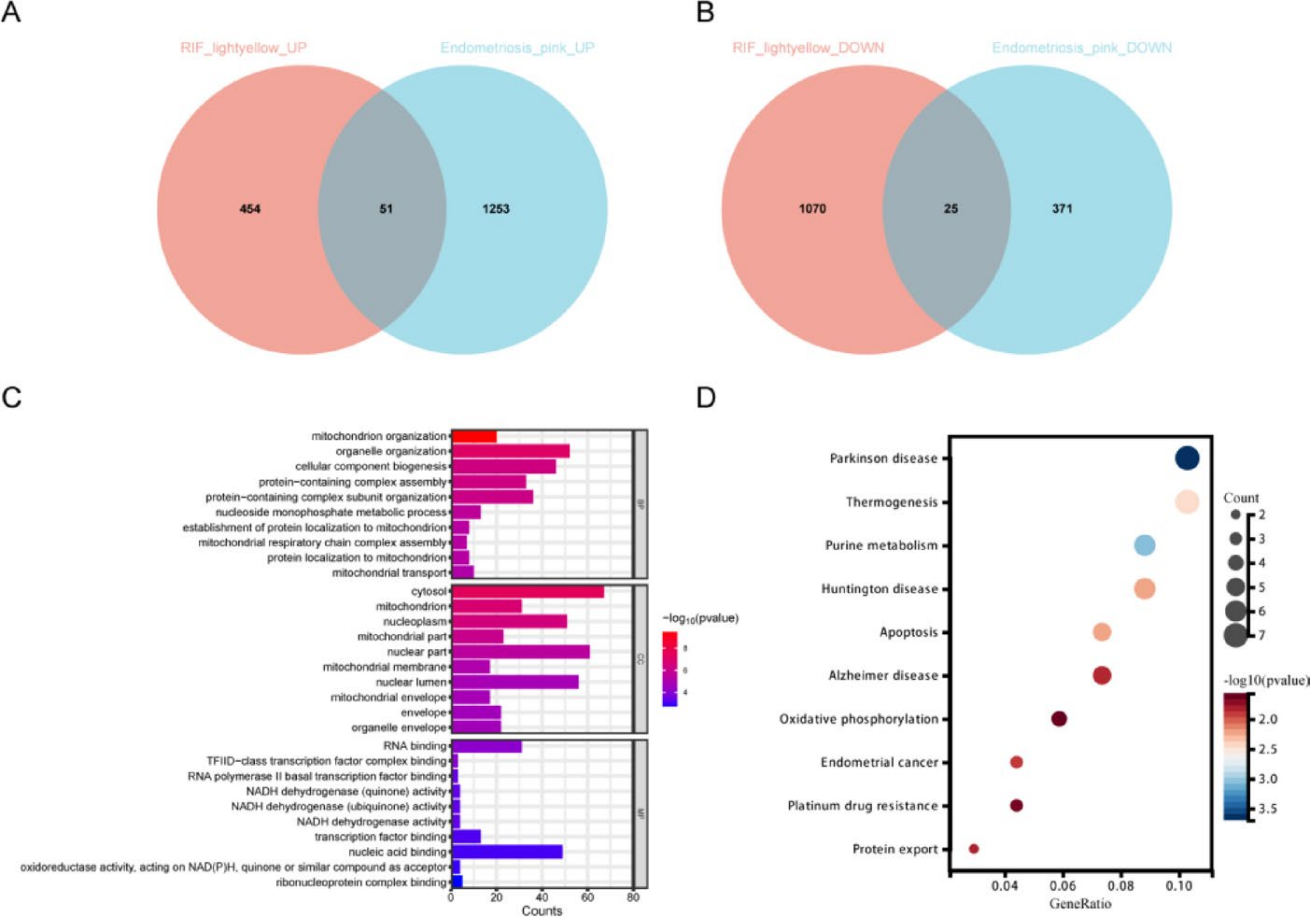
Acquisition and functional enrichment analysis of shared susceptibility genes from endometriosis and RIF. (**A**) Venn diagram of up-regulated genes in key modules associated with endometriosis and RIF. (**B**) Venn diagram of down-regulated genes in key modules associated with endometriosis and RIF. (**C**) GO functional enrichment analysis of shared susceptibility genes in the Venn diagram. The top 10 enrichment results of BPs, MFs, and CCs in GO, respectively. (**D**) The top 10 KEGG pathways of shared susceptibility genes in the Venn diagram.

Based on the WGCNA analysis results, the intersections of upregulated and downregulated genes between endometriosis-related module genes and RIF-related module genes were taken, yielding 51 upregulated intersecting genes (Fig. 3A) and 25 downregulated intersecting genes (Fig. 3B). Combining these genes resulted in a total of 76 shared susceptibility genes. Subsequently, the functions of these genes were explored through GO and KEGG analyses. The enrichment analysis indicated that these genes were significantly enriched in 595 GO terms, including 445 BPs such as mitochondrion organization, organelle organization, and mitochondrial transport; 87 CCs such as cytosol, mitochondrion, and nucleoplasm; and 63 MFs such as RNA binding, NADH dehydrogenase activity, and oxidoreductase activity (Fig. 3C). In the KEGG pathway analysis, these genes were primarily enriched in 19 pathways, including apoptosis, oxidative phosphorylation, and endometrial cancer (Fig. 3D).

### Evaluation of expression patterns and diagnostic efficacy of hub genes in endometriosis and RIF

**Fig. 4:**
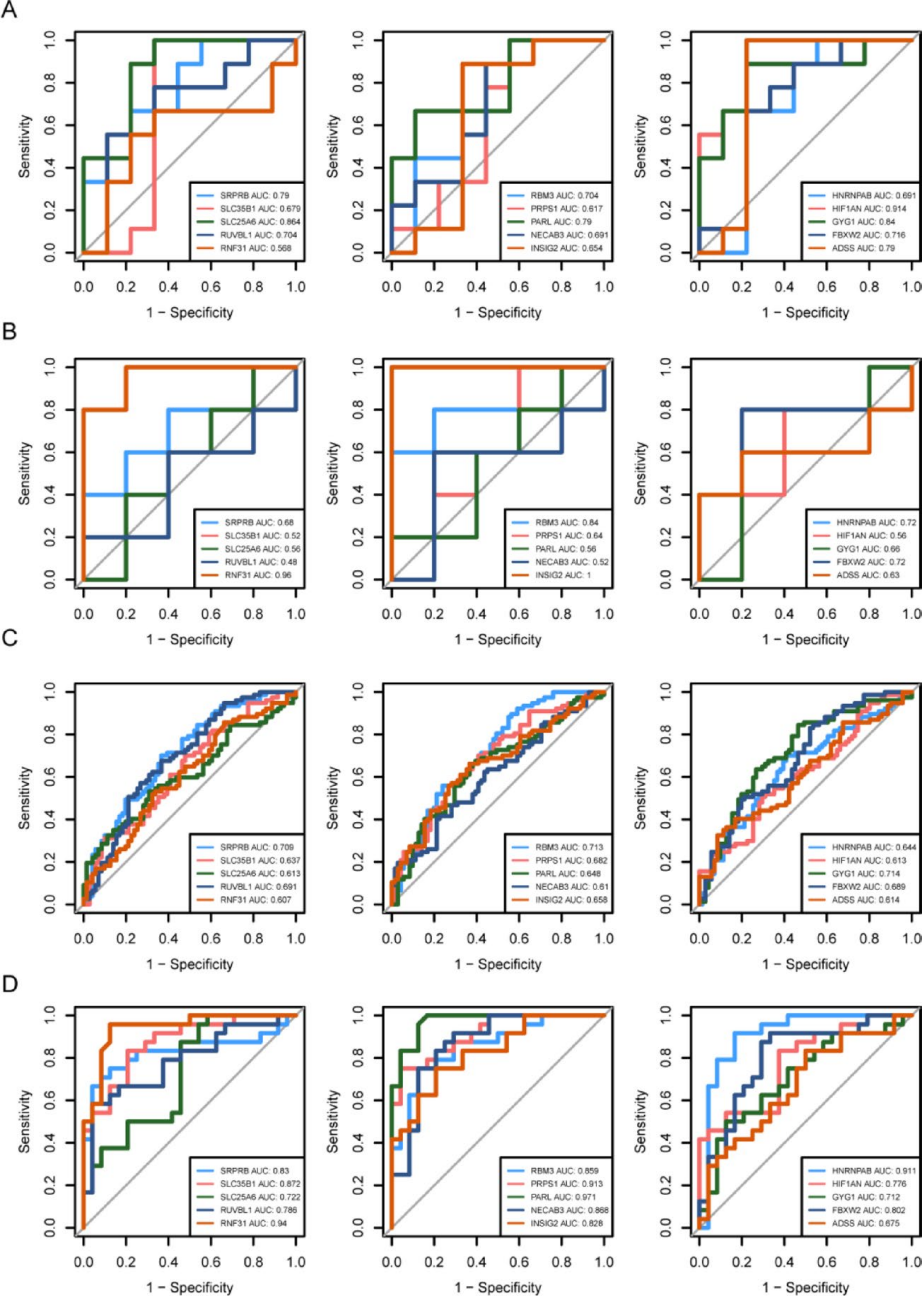
ROC curve analysis of hub genes to evaluate diagnostic accuracy and identify diagnostic genes for endometriosis and RIF. (**A**) ROC curves of 15 hub genes in the GSE25628 training set of endometriosis. (**B**) ROC curves of 15 hub genes in the GSE51981 validation set of endometriosis. (**C**) ROC curves of 15 hub genes in the GSE111974 training set of RIF. (**D**) ROC curves of 15 hub genes in the GSE26787 validation set of RIF.

To investigate the expression patterns and the discriminative ability of the 76 shared susceptibility genes between disease and normal samples, differential expression analysis was first conducted on these genes in the training and validation sets of endometriosis and RIF. The results showed that 15 genes exhibited consistent up/down-regulation trends with significant differences (*p* < 0.05) in both the training and validation sets: SRPRB, SLC35B1, SLC25A6, RUVBL1, RNF31, RBM3, PRPS1, PARL, NECAB3, INSIG2, HNRNPAB, HIF1AN, GYG1, FBXW2, and ADSS (Supplementary Table S2). Further ROC analysis revealed that among these 15 hub genes, SRPRB, RBM3, INSIG2, GYG1, and FBXW2 exhibited AUC values greater than 0.65 in all four datasets, indicating high diagnostic accuracy (Fig. 4A–D). This suggests that these five genes possess strong discriminative power and can serve as diagnostic genes for endometriosis and RIF patients.

### Immune cell infiltration and its relevance with diagnostic genes

**Fig. 5:**
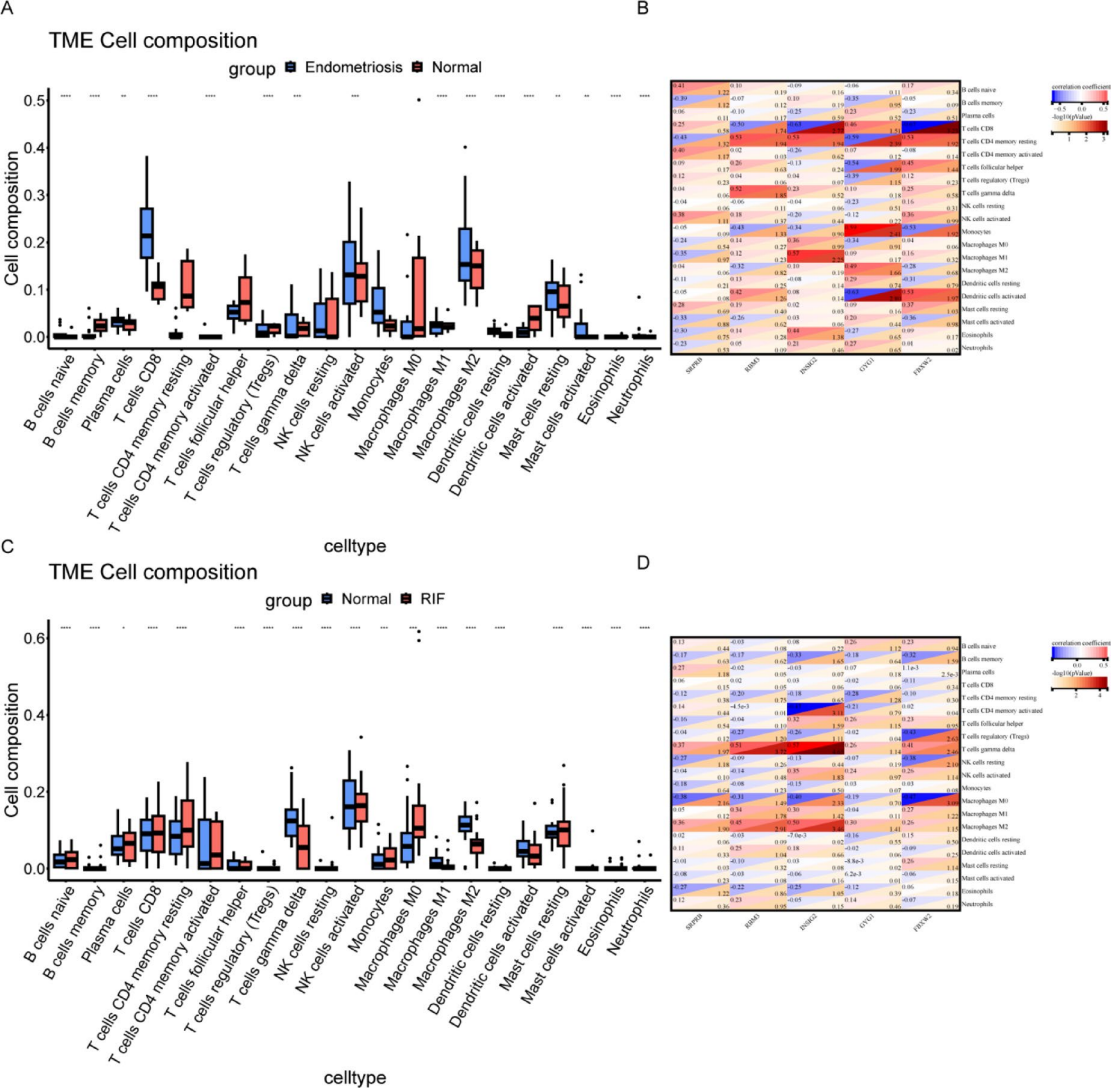
Results of immune infiltration analysis of endometriosis and RIF. (**A**) Comparison of 22 immune cell type differences between the endometriosis group and the normal group using the CIBERSORT algorithm. (**B**) Heatmap of correlations between diagnostic genes and immune cells in endometriosis. (**C**) Comparison of 22 immune cell type differences between the normal group and the RIF group using the CIBERSORT algorithm. (**D**) Heatmap of correlations between diagnostic genes and immune cells in RIF. *p < 0.05; **p < 0.01; ***p< 0.001; ****p < 0.0001.

Immune infiltration analysis was conducted on the training set of endometriosis, utilizing the CIBERSORT algorithm to evaluate the proportions of 22 immune cell types across various samples. As depicted in Figs. 5A and 16 immune cell types exhibited significant differences between the endometriosis group and the normal control group. The infiltration abundance of B cells naive, plasma cells, T cells CD8, T cells CD4 memory activated, natural killer (NK) cells activated, macrophages M1, macrophages M2, dendritic cells resting, mast cells resting, mast cells activated, and neutrophils was significantly higher in the endometriosis group than in the normal control group. In contrast, B cells memory, T cells regulatory (Tregs), T cells gamma delta, dendritic cells activated, and eosinophils exhibited the opposite trend. The correlation analysis between immune cells and diagnostic genes revealed that GYG1 had the highest positive correlation with monocytes (cor = 0.59, -log10(pValue) = 2.41), followed by INSIG with Macrophages M1 (cor = 0.57, -log10(pValue) = 2.25). Notably, the strong negative correlation between FBXW2 and CD8⁺ T cells (cor = −0.67,-log10(pValue) = 3.23) suggests CD8⁺ T cell-mediated inflammatory microenvironments may downregulate this tumor suppressor gene during endometrial remodeling (PMID: 39370077).GYG1 also showed significant negative correlation with activated dendritic cells (cor = −0.63, -log10(pValue) = 2.80) (Fig. 5B).

Analysis of the recurrent implantation failure (RIF) training set identified 19 differentially infiltrated immune cells (Fig. 5C). RIF samples exhibited elevated levels of B cells naive, plasma cells, CD8⁺ T cells, activated NK cells, monocytes, M0 macrophages, and resting mast cells, whereas controls showed higher γδ T cells and M1/M2 macrophages. Intriguingly, INSIG2 displayed the strongest positive correlation with γδ T cells (cor = 0.57, -log10(pValue) = 4.65), which typically exhibit immunosuppressive functions in reproductive tissues. RBM3 also positively correlated with γδ T cells (cor = 0.51, -log10(pValue) = 3.72). For negatively correlated pairs, INSIG2 and activated CD4⁺ memory T cells (cor = −0.47, -log10(pValue) = 3.11) showed the strongest inverse relationship, implying Th1-type inflammation may suppress INSIG2-mediated lipid metabolism in the implantation window. FBXW2 and M0 macrophages showed comparable negative correlation (cor = −0.47, -log10(pValue) = 3.09) (Fig. 5D). Collectively, these immune-gene correlations delineate shared regulatory circuits between endometriosis and RIF, particularly highlighting CD8⁺ T cell and macrophage-driven mechanisms that disrupt endometrial homeostasis through inflammatory and metabolic reprogramming pathways.

### Expression of diagnostic genes at the single cell level

**Fig. 6:**
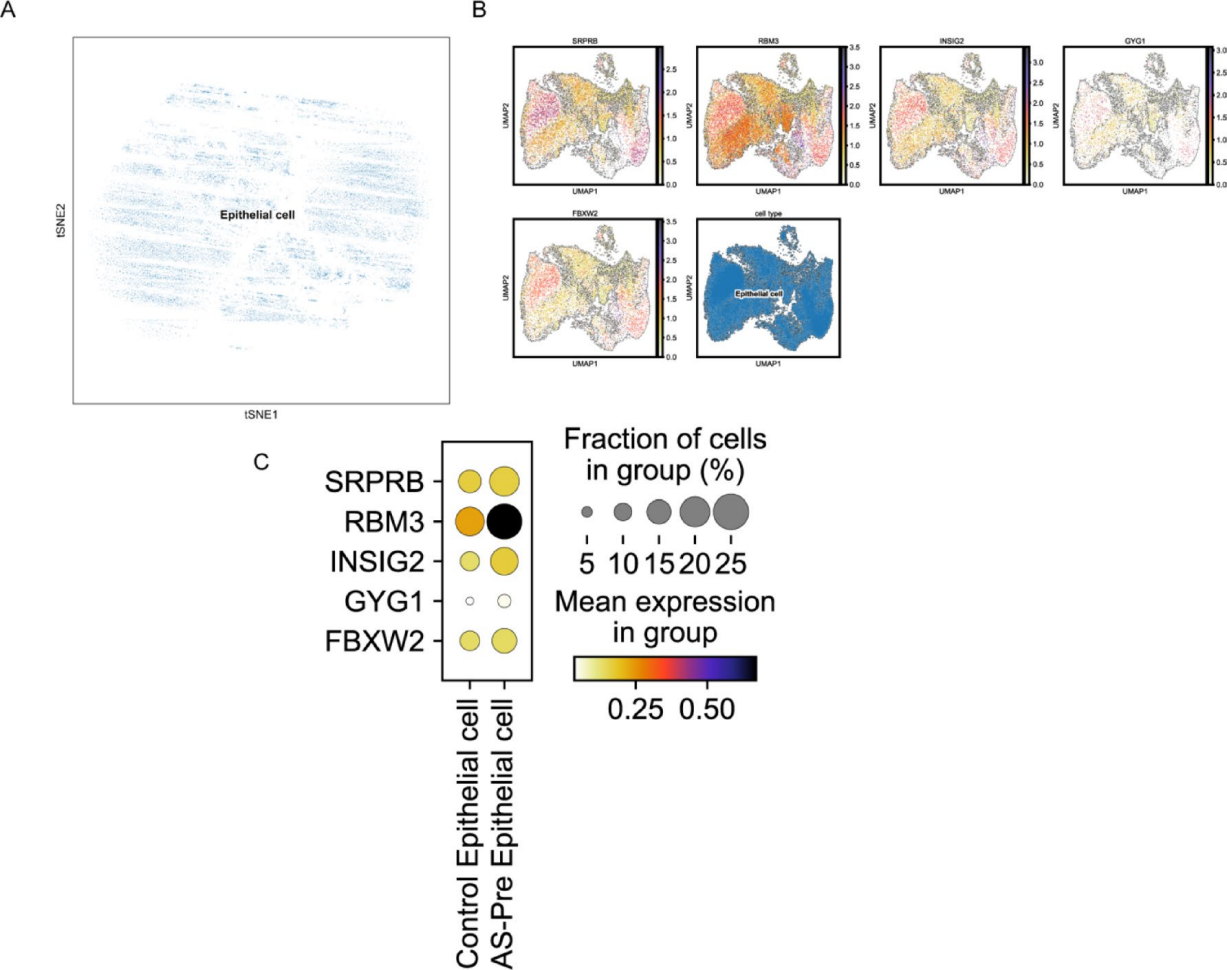
Expression levels of diagnostic genes in scRNA-seq dataset GSE216748. (**A**) Clustering of epithelial cells in the endometrial organoids. (**B**) Expression of five diagnostic genes in epithelial cells. (**C**) Expression of five diagnostic genes in epithelial cells between control and AS pre-treatment groups.

To investigate the expression of diagnostic genes at the single-cell level, the scRNA-seq dataset GSE216748 was downloaded for analysis. After quality control, dimensionality reduction, and clustering, a single cell cluster was obtained, identified as epithelial cells (Fig. 6A). Subsequently, using the Seurat package, five diagnostic genes (SRPRB, RBM3, INSIG2, GYG1, and FBXW2) were mapped onto the UMAP plot. It was observed that these genes were evenly distributed within the epithelial cells, with RBM3 exhibiting relatively higher expression levels in the epithelial cells (Fig. 6B). Furthermore, the expression differences of diagnostic genes between control epithelial cells and AS-Pre epithelial cells were explored. The results indicated that the expression of RBM3 was higher in AS-Pre epithelial cells compared to control epithelial cells (Fig. 6C).

### Expression validation of five diagnostic genes in tissues

**Fig. 7:**
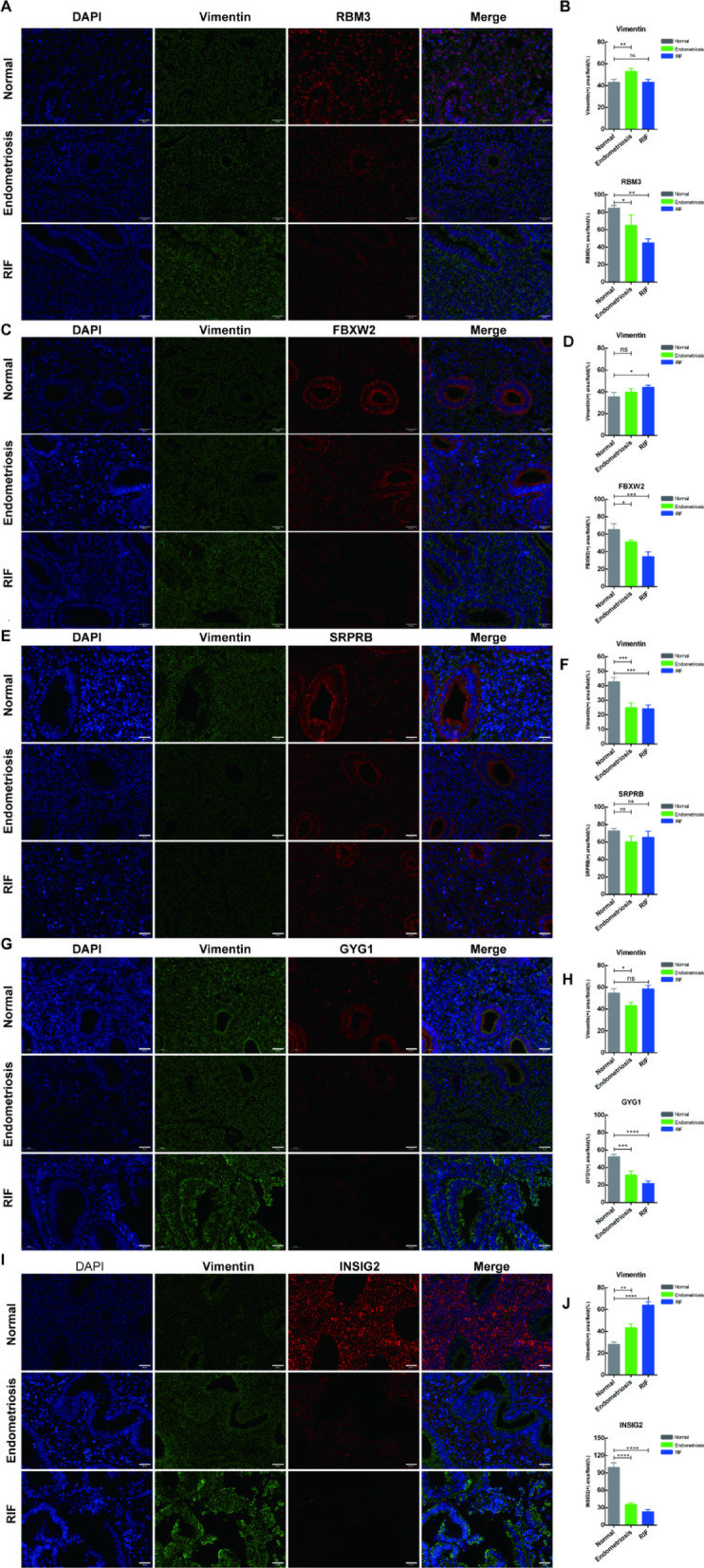

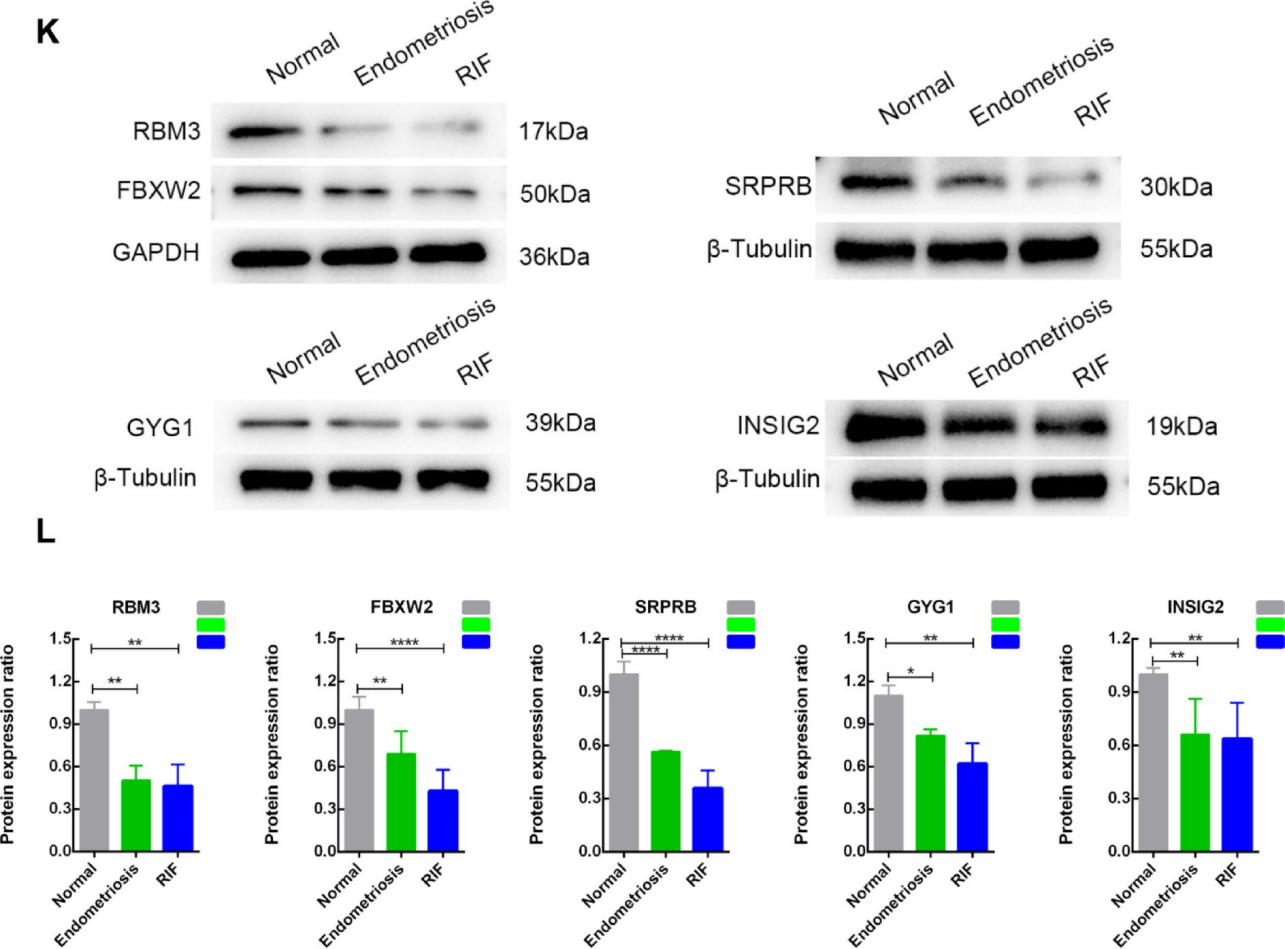
Expression of diagnostic genes were validated between normal control, endometriosis, and RIF groups. (**A**–**J**). Immunofluorescent staining and quantitative analysis of RBM3 (**A**, **B**), FBXW2 (**C**, **D**), SRPRB (**E**, **F**), GYG1 (**G**, **H**), and INSIG2 (**I**, **J**). Scale bar = 25 μm. The target proteins exhibited red fluorescence, Vimentin showed green fluorescence, and the nuclei were counterstained with blue. (**K**, **L**) Western blotting and quantitative analysis of RBM3, FBXW2, SRPRB, GYG1, and INSIG2. Data are expressed as mean ± standard deviation. Normal control and RIF groups, *n* = 12; endometriosis groups, *n* = 9. ns, no significance; *p < 0.05;** *p* < 0.01; ***p < 0.001; ****p < 0.0001.

To further validate the actual expression levels of the five diagnostic genes in endometriosis and RIF, we performed immunofluorescence staining and Western blot analysis on endometrial tissues from normal, endometriosis, and RIF groups.Immunofluorescence double staining showed that the fluorescence signal intensities of SRPRB, RBM3, INSIG2, GYG1, and FBXW2 (red fluorescence) were significantly decreased in both disease groups compared to the normal group, while Vimentin expression (green fluorescence) was also generally reduced in endometriosis and RIF groups (Fig. 7A,C,E,G,I). The corresponding statistical analyses further confirmed these observations (Fig. 7B,D,F,H,J).

Western blot analysis consistently demonstrated that the protein expression levels of SRPRB, RBM3, INSIG2, GYG1, and FBXW2 were significantly reduced in both endometriosis and RIF groups compared to the normal group (Fig. 7K), with statistical analysis validating these findings (Fig. 7L).

These results align with our bioinformatics analysis, indicating that these diagnostic genes are indeed dysregulated in endometriosis and RIF endometrial tissues. The proteins they encode may play crucial roles in regulating endometrial receptivity.

## Discussion

In recent years, assisted reproductive technology (ART) has significantly improved pregnancy rates, yet RIF continues to affect approximately 10% of patients undergoing treatment^3^. RIF is clinically characterized by the failure to achieve pregnancy after repeated transfers of high-quality embryos into a normal uterus, though its definition remains heterogeneous across studies^14^. Notably, a subset of RIF patients present without apparent clinical abnormalities, suggesting underlying undiagnosed conditions such as endometriosis - a systemic inflammatory disorder known to impair fertility outcomes^28,29^. This study employed an integrated multi-omics approach to identify and validate five core molecules (*SRPRB*,* RBM3*,* INSIG2*,* GYG1*,* and FBXW2*) demonstrating significant differential expression in both endometriosis and RIF, with robust diagnostic discriminatory power. Our comprehensive analysis extends beyond mere identification, revealing these hub genes as central players in the intricate interplay between endometrial function, immune regulation, and metabolic homeostasis.

*SRPRB* (Signal Recognition Particle Receptor B) functions as a critical component of the protein targeting machinery to the endoplasmic reticulum. Its dysregulation potentially disrupts the secretion of essential implantation mediators, including cytokines, growth factors, and adhesion molecules, thereby compromising the endometrial preparation for embryo attachment^30^. *RBM3* (RNA Binding Motif Protein 3), an RNA-binding protein and cold-shock protein, serves as a post-transcriptional regulator during the implantation window. Its diminished expression likely impairs the stability and translation of mRNAs crucial for embryo adhesion and stromal cell decidualization, effectively disrupting the molecular orchestration of endometrial receptivity^31^. *INSIG2* (Insulin Induced Gene 2) emerges as a pivotal metabolic-immune integrator. As a key regulator of cholesterol and lipid metabolism, its dysregulation alters the local lipid milieu, creating a pro-inflammatory environment that recruits monocytes and macrophages while simultaneously disrupting the metabolic preparedness required for successful implantation^32^. *GYG1* (Glycogenin 1) plays an essential role in glycogen biosynthesis, highlighting the underappreciated importance of bioenergetic preparation for receptivity. Aberrant *GYG1* expression may create metabolic deficiencies, depriving the endometrium of glycogen-derived energy necessary for the energetically demanding processes of embryo attachment and stromal decidualization^33^. *FBXW2* (F-Box and WD Repeat Domain Containing 2), an E3 ubiquitin ligase subunit, maintains protein homeostasis by targeting key signaling molecules for degradation. Its suppression, potentially mediated by inflammatory cytokines, could lead to aberrant accumulation of proteins involved in critical pathways such as Wnt/β-catenin signaling, thereby disrupting endometrial homeostasis^34^.

Our immune infiltration analysis reveals a sophisticated gene-immune interactome that actively drives endometrial pathology in both conditions. The most striking shared immune feature is the significant elevation of CD8⁺ T cells and macrophage subsets, indicating a persistent cytotoxic inflammatory environment^35,36^.

The strong negative correlation between *FBXW2* and CD8⁺ T cells suggests a direct mechanistic relationship where CD8⁺ T cell-derived cytokines (particularly IFN-γ) may transcriptionally suppress this tumor suppressor gene. This suppression potentially removes critical brakes on cellular proliferation while simultaneously fostering a pro-inflammatory milieu conducive to disease progression^37^. Conversely, the positive correlation between *INSIG2* and monocyte/macrophage infiltration establishes its role at the nexus of metabolism and innate immunity. *INSIG2* dysregulation may generate lipid-based “find-me” signals that perpetuate immune cell recruitment and activation, creating a self-sustaining inflammatory cycle^38^. The positive association between *RBM3* and γδ T cells in RIF may represent a failed compensatory immunosuppressive mechanism that becomes overwhelmed by the overarching inflammatory environment^5^.

These findings extend beyond those of Salmeri et al.^39^ by not only validating immune cell involvement but also mapping specific immune populations to hub gene activity, suggesting these genes function as active regulators rather than passive biomarkers within the immunopathological landscape.

The consistent diagnostic performance (AUC > 0.65) of our five-gene signature supports its development into a molecular diagnostic panel for objective assessment of endometrial receptivity. This multi-gene approach could be implemented as a minimally invasive endometrial biopsy test to identify receptivity defects, particularly in cases of idiopathic RIF where occult endometriosis is suspected^40^.

The distinct gene-immune interaction patterns suggest compelling avenues for personalized therapeutic strategies. Patients exhibiting the “FBXW2-low/CD8⁺ T cell-high” signature might benefit from immunomodulatory interventions targeting cytotoxic T cell responses. Those with the “INSIG2-high/monocyte-high” signature could be candidates for therapies targeting metabolic-inflammatory cross-talk. Prospective studies validating this gene signature’s ability to predict IVF outcomes could enable risk stratification and personalized intervention plans, potentially including embryo banking, targeted immune therapy, or surgical investigation for endometriosis prior to undergoing multiple failed IVF cycles.

While our sample size proved sufficient for this discovery-phase multi-omics study, we acknowledge the necessity for validation in larger, multi-center cohorts to ensure generalizability. Future work will include power calculations and prospective recruitment to solidify these findings.

The correlative nature of our current findings necessitates rigorous functional validation through in vitro and in vivo models. Future experiments will examine whether CD8⁺ T cell conditioned medium suppresses *FBXW2* expression in endometrial cells and how *INSIG2* overexpression alters monocyte recruitment and polarization. Longitudinal sampling across the menstrual cycle will be essential to capture the dynamic expression patterns of these genes and their associated immune cells, adding a crucial temporal dimension to our current understanding^41^.

Despite these limitations, our work provides a robust foundation for understanding the molecular mechanisms underlying endometrial receptivity disorders and offers concrete pathways toward advancing individualized precision medicine in reproductive health.

## Supplementary Information

Below is the link to the electronic supplementary material.

Supplementary Material 1

Supplementary Material 2

Supplementary Material 3

Supplementary Material 4

Supplementary Material 5

Supplementary Material 6

Supplementary Material 7

Supplementary Material 8

Supplementary Material 9

Supplementary Material 10

Supplementary Material 11

Supplementary Material 12

Supplementary Material 13

Supplementary Material 14

Supplementary Material 15

Supplementary Material 16

Supplementary Material 17

Supplementary Material 18

Supplementary Material 19

Supplementary Material 20

Supplementary Material 21

Supplementary Material 22

Supplementary Material 23

Supplementary Material 24

Supplementary Material 25

Supplementary Material 26

Supplementary Material 27

Supplementary Material 28

Supplementary Material 29

Supplementary Material 30

Supplementary Material 31

Supplementary Material 32

## Data Availability

Data is provided within the manuscript or supplementary information files.
