# Supplementary material for: Multi-omics analysis reveals shared diagnostic and therapeutic targets in endometriosis and recurrent implantation failure
Source: Sci Rep. 2025 Dec 29;15:44874. doi: 10.1038/s41598-025-28877-8 (PMC12748698; doi:10.1038/s41598-025-28877-8)
Supplement: Supplementary file 1 — Supplementary Material 1 [file 41598_2025_28877_MOESM1_ESM.docx]

**Supplementary Tables**

**Table S1. Comparison of general information of the three groups of patients.**

| **Patients’ characteristics** | **Normal control group**  **(n = 12)** | **Endometriosis group**  **(n = 9)** | **RIF group (n = 12)** | ***P*** |
| --- | --- | --- | --- | --- |
| Age (year) | **29.67±0.333** | **29.44±0.412** | **29.75±0.775** | **0.93** |
| BMI (kg/m²) | **22.825±1.333** | **22.76±0.834** | **23.24±1.100** | **0.56** |
| Gravidity | **1±0** | **1±0** | **3±0.778** | **< 0.01** |
| Menstrual average cycle (days) | **31.5±5.95** | **32.44±4.43** | **30.08±5.24** | **0.594** |
| Menstrual duration (days) | **5.5±2.00** | **9±1.88** | **6.15±1.77** | **0.37** |

**Notes:** Data are shown as the mean ± standard deviation. Significant difference at *p* < 0.05.

**Abbreviations:** BMI, body mass index; RIF, recurrent implantation failure.

**Table S2. Validation of hub gene expression in endometriosis (EM) and recurrent implantation failure (RIF).**

|  | EM training set GSE25628 | | EM validation set GSE51981 | | RIF training set [GSE111974](https://www.ncbi.nlm.nih.gov/geo/query/acc.cgi?acc=GSE111974) | | RIF validation set [GSE26787](https://www.ncbi.nlm.nih.gov/geo/query/acc.cgi?acc=GSE26787) | |
| --- | --- | --- | --- | --- | --- | --- | --- | --- |
| Gene | P.Value | logFC | P.Value | logFC | P.Value | logFC | P.Value | logFC |
| SRPRB | 0.024724242 | -0.561726538 | 0.0000124 | -0.495922169 | 0.0000397 | -0.445573377 | 0.01824887 | -289.03575 |
| SLC35B1 | 0.024730621 | -0.519336862 | 0.006137224 | -0.280891085 | 0.00000814 | -0.529041667 | 0.01289126 | -518.758525 |
| SLC25A6 | 0.000212446 | 0.601664461 | 0.036226814 | 0.203423076 | 0.003117326 | -0.259395833 | 0.000485148 | -1228.09525 |
| RUVBL1 | 0.001026787 | -0.800137843 | 0.000113572 | -0.502268042 | 0.000574149 | -0.5977125 | 0.018410128 | -77.30698681 |
| RNF31 | 0.0000352 | 0.898505025 | 0.02105809 | 0.161917058 | 0.00000312 | 0.64835 | 0.04837998 | 119.5879825 |
| RBM3 | 0.006693996 | -0.897252133 | 0.00000632 | -0.64679009 | 0.000000921 | -0.862783333 | 0.004213301 | -278.6828525 |
| PRPS1 | 0.001685672 | -0.589809141 | 0.0000888 | -0.446921155 | 5.75E-08 | -0.575071457 | 0.019735034 | -182.538325 |
| PARL | 0.00000912 | -0.575838983 | 0.009118428 | -0.19120856 | 1.66E-11 | -0.659504167 | 0.003667428 | -179.2161767 |
| NECAB3 | 0.002076903 | 0.784419954 | 0.010938311 | 0.444869732 | 0.00000423 | 0.531841667 | 0.007137028 | 13.51368311 |
| INSIG2 | 0.003502166 | -0.911794999 | 0.000399103 | -0.635815286 | 0.0000256 | -0.6573625 | 0.0000588 | -203.01026 |
| HNRNPAB | 0.010683708 | -0.693435553 | 0.004350754 | -0.236412879 | 0.000000324 | -1.028537113 | 0.021067373 | -544.5350875 |
| HIF1AN | 1.64E-08 | -0.992843246 | 0.005031973 | -0.168774087 | 0.000430461 | -0.3340375 | 0.002045579 | -79.9010925 |
| GYG1 | 0.000164127 | -0.807040963 | 0.000240513 | -0.461261994 | 0.022249944 | -0.176975 | 0.011975506 | -362.62045 |
| FBXW2 | 0.000544971 | -0.651557362 | 0.000057 | -0.359737372 | 0.000395223 | -0.459132926 | 0.000582203 | -55.61355375 |
| ADSS | 5.31E-08 | -2.171455252 | 0.003966742 | -0.255783845 | 0.019652109 | -0.29025 | 0.019488743 | -267.9291888 |
