# Supplementary figures and images for: Multi-omics analysis reveals shared diagnostic and therapeutic targets in endometriosis and recurrent implantation failure
Source: Sci Rep. 2025 Dec 29;15:44874. doi: 10.1038/s41598-025-28877-8 (PMC12748698; doi:10.1038/s41598-025-28877-8)

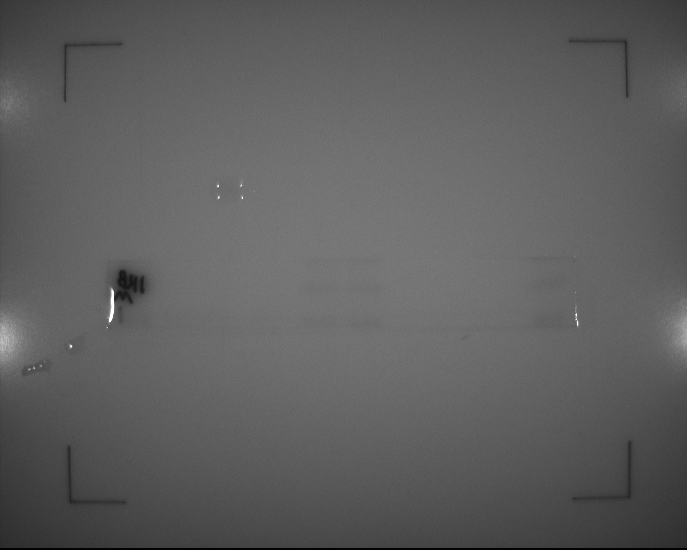

Supplement: Supplementary file 2 — Supplementary Material 2 [file 41598_2025_28877_MOESM2_ESM.tif]

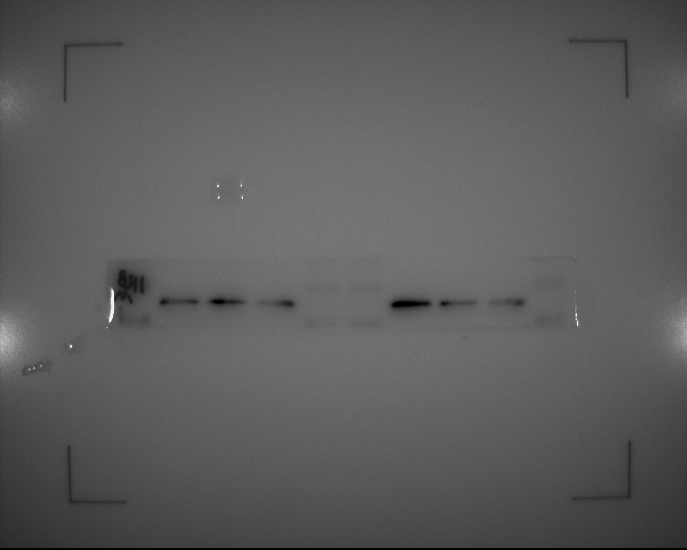

Supplement: Supplementary file 3 — Supplementary Material 3 [file 41598_2025_28877_MOESM3_ESM.tif]

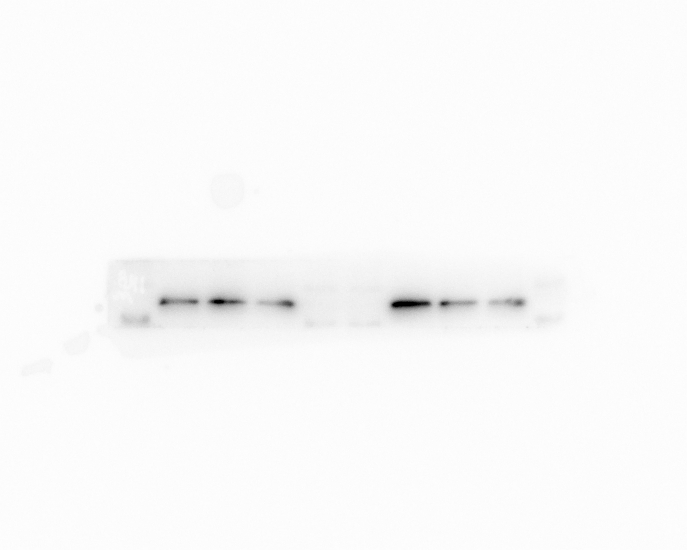

Supplement: Supplementary file 4 — Supplementary Material 4 [file 41598_2025_28877_MOESM4_ESM.tif]

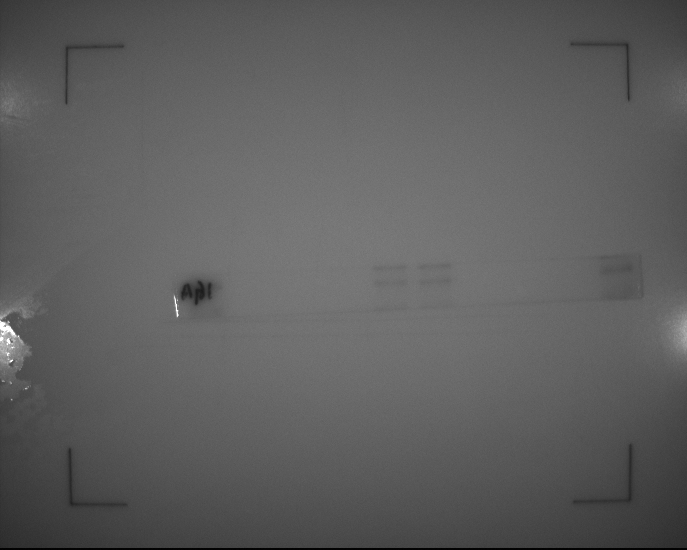

Supplement: Supplementary file 5 — Supplementary Material 5 [file 41598_2025_28877_MOESM5_ESM.tif]

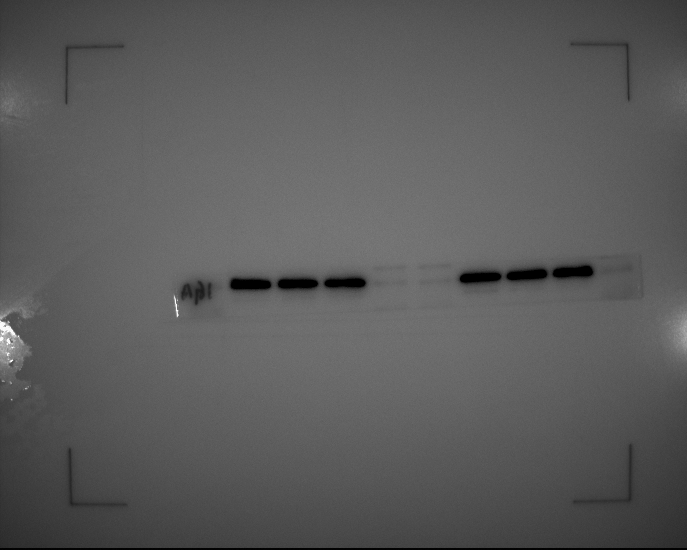

Supplement: Supplementary file 6 — Supplementary Material 6 [file 41598_2025_28877_MOESM6_ESM.tif]

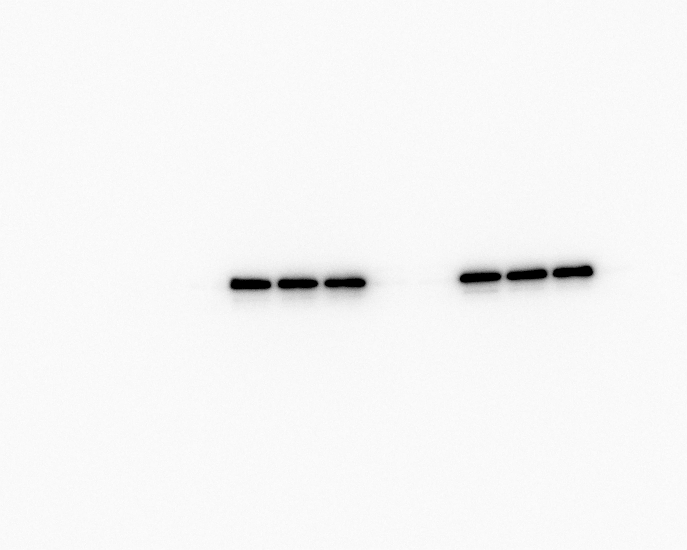

Supplement: Supplementary file 7 — Supplementary Material 7 [file 41598_2025_28877_MOESM7_ESM.tif]

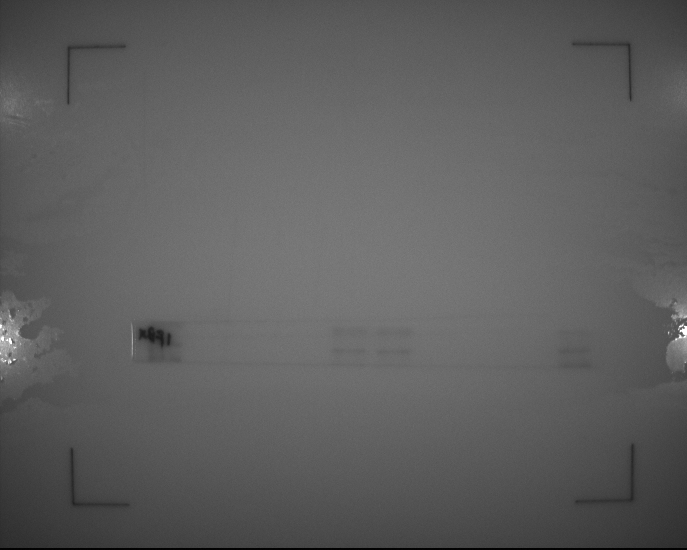

Supplement: Supplementary file 8 — Supplementary Material 8 [file 41598_2025_28877_MOESM8_ESM.tif]

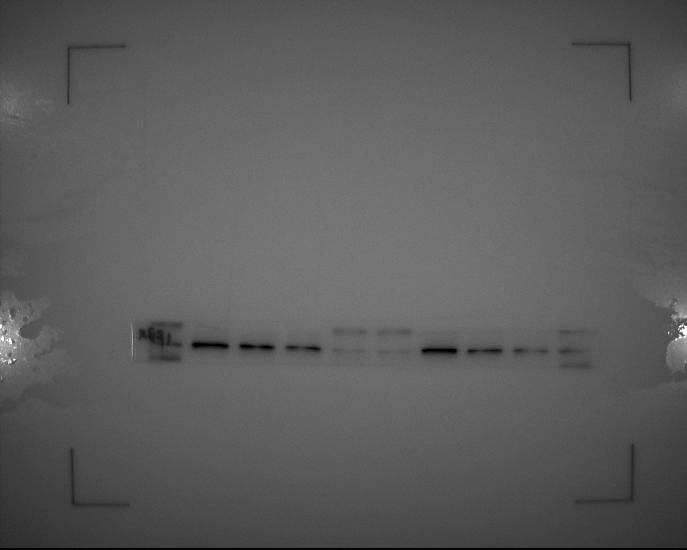

Supplement: Supplementary file 9 — Supplementary Material 9 [file 41598_2025_28877_MOESM9_ESM.tif]

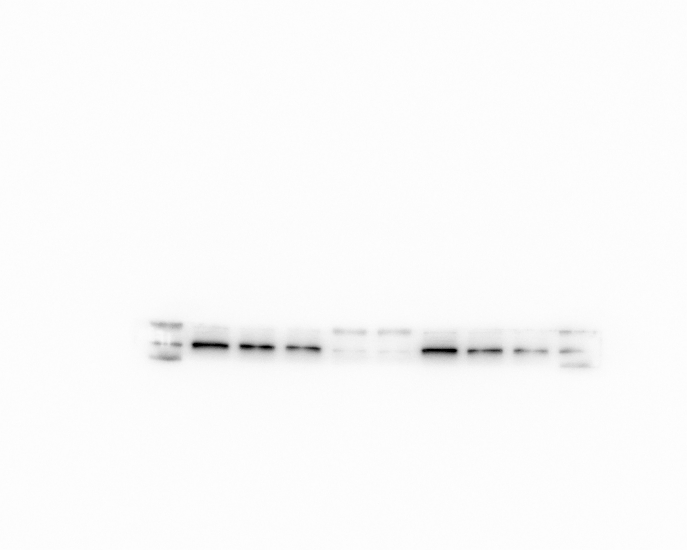

Supplement: Supplementary file 10 — Supplementary Material 10 [file 41598_2025_28877_MOESM10_ESM.tif]

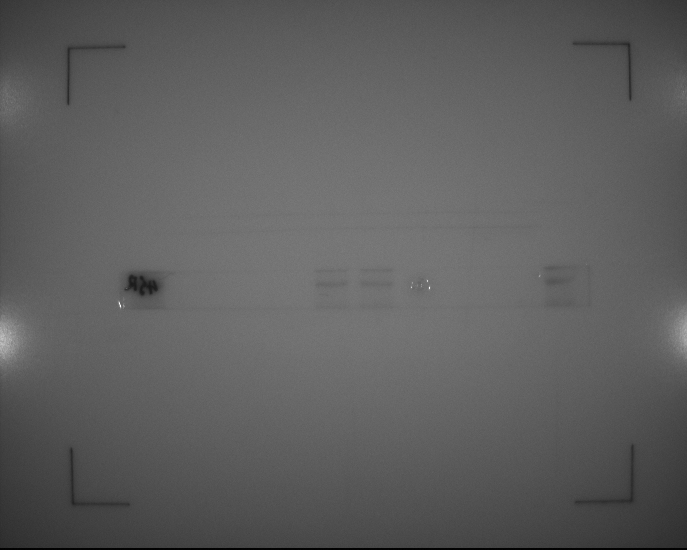

Supplement: Supplementary file 14 — Supplementary Material 14 [file 41598_2025_28877_MOESM14_ESM.tif]

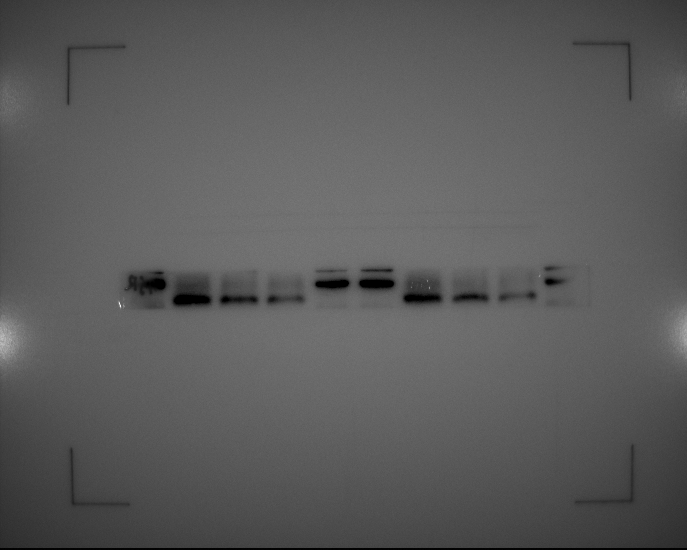

Supplement: Supplementary file 15 — Supplementary Material 15 [file 41598_2025_28877_MOESM15_ESM.tif]

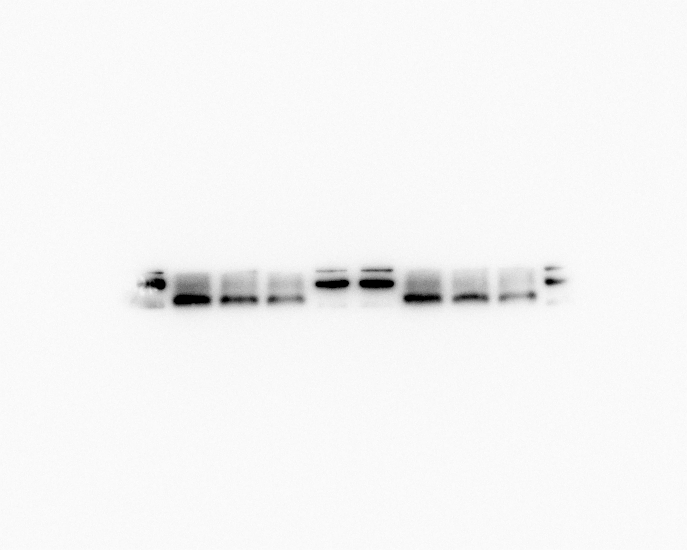

Supplement: Supplementary file 16 — Supplementary Material 16 [file 41598_2025_28877_MOESM16_ESM.tif]

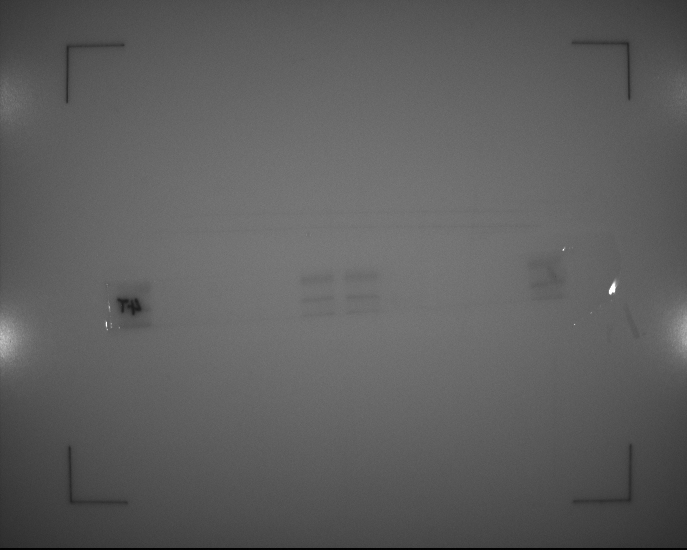

Supplement: Supplementary file 17 — Supplementary Material 17 [file 41598_2025_28877_MOESM17_ESM.tif]

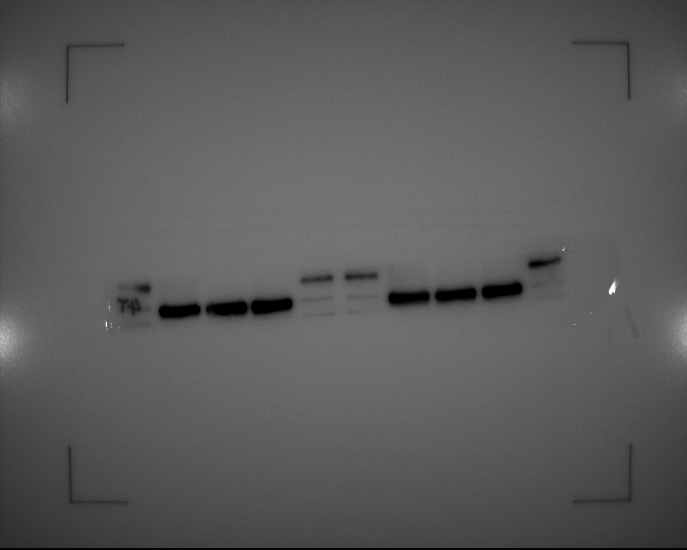

Supplement: Supplementary file 18 — Supplementary Material 18 [file 41598_2025_28877_MOESM18_ESM.tif]

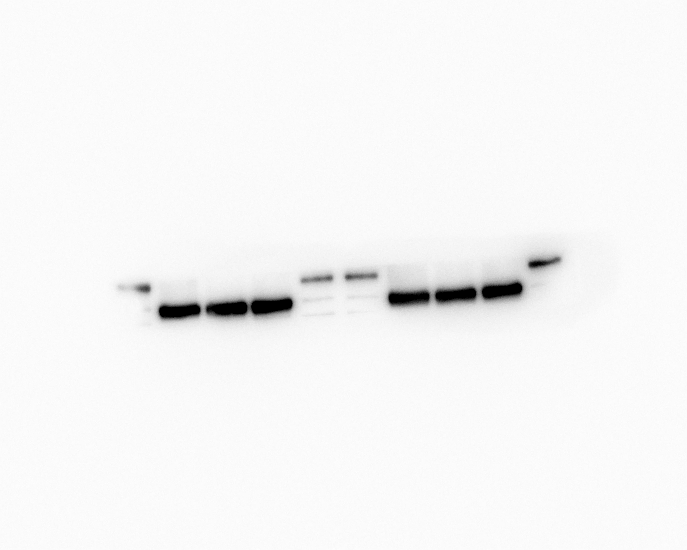

Supplement: Supplementary file 19 — Supplementary Material 19 [file 41598_2025_28877_MOESM19_ESM.tif]

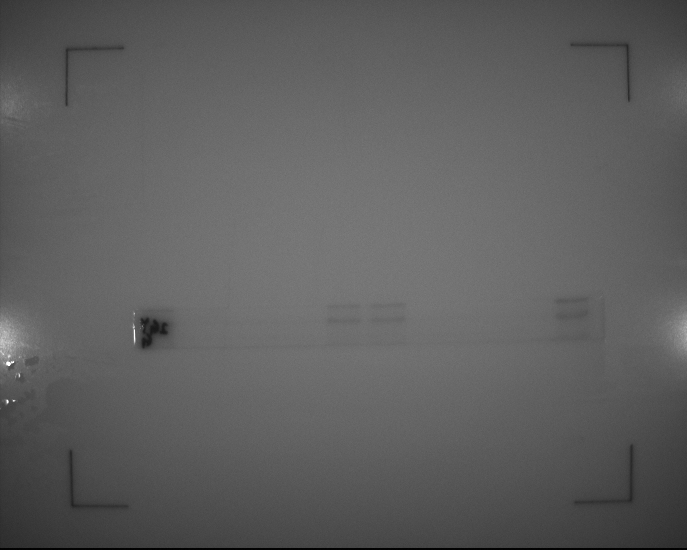

Supplement: Supplementary file 20 — Supplementary Material 20 [file 41598_2025_28877_MOESM20_ESM.tif]

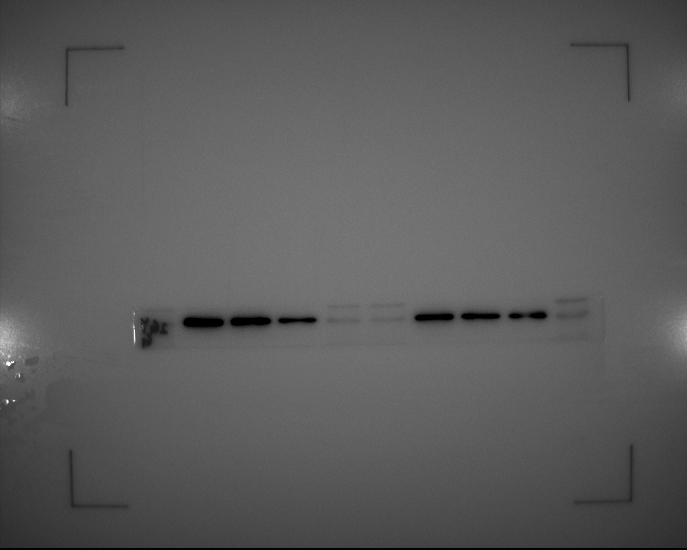

Supplement: Supplementary file 21 — Supplementary Material 21 [file 41598_2025_28877_MOESM21_ESM.tif]

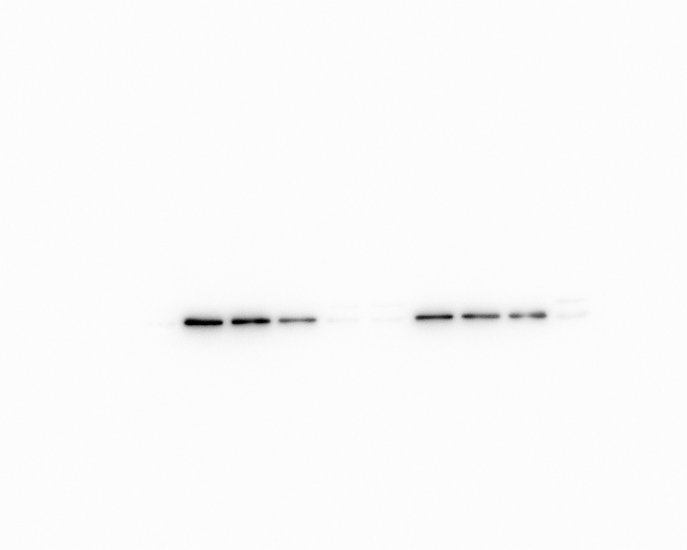

Supplement: Supplementary file 22 — Supplementary Material 22 [file 41598_2025_28877_MOESM22_ESM.tif]

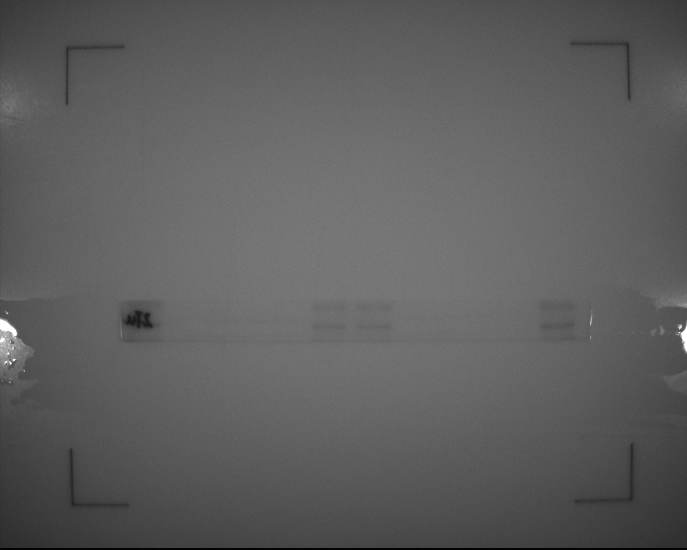

Supplement: Supplementary file 23 — Supplementary Material 23 [file 41598_2025_28877_MOESM23_ESM.tif]

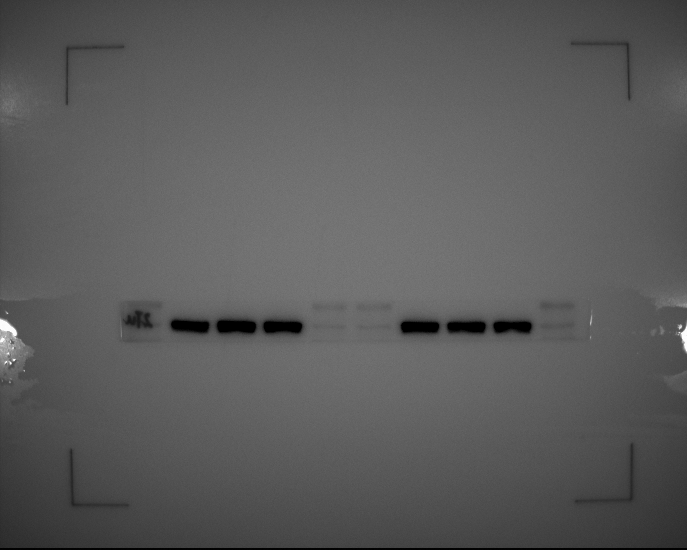

Supplement: Supplementary file 24 — Supplementary Material 24 [file 41598_2025_28877_MOESM24_ESM.tif]

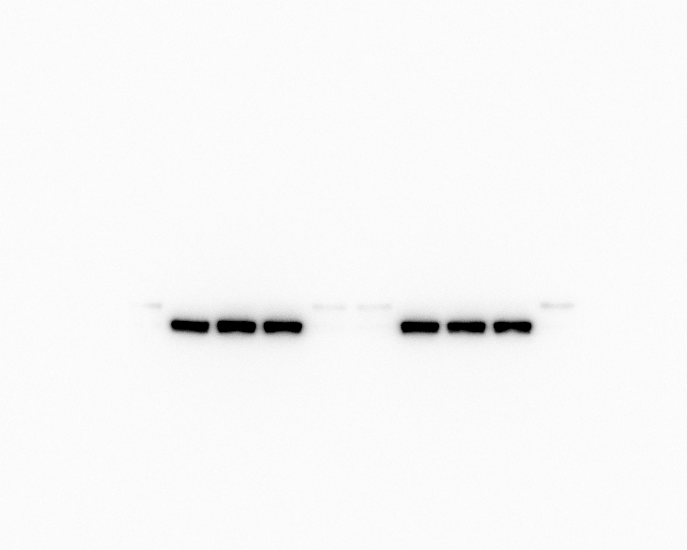

Supplement: Supplementary file 25 — Supplementary Material 25 [file 41598_2025_28877_MOESM25_ESM.tif]

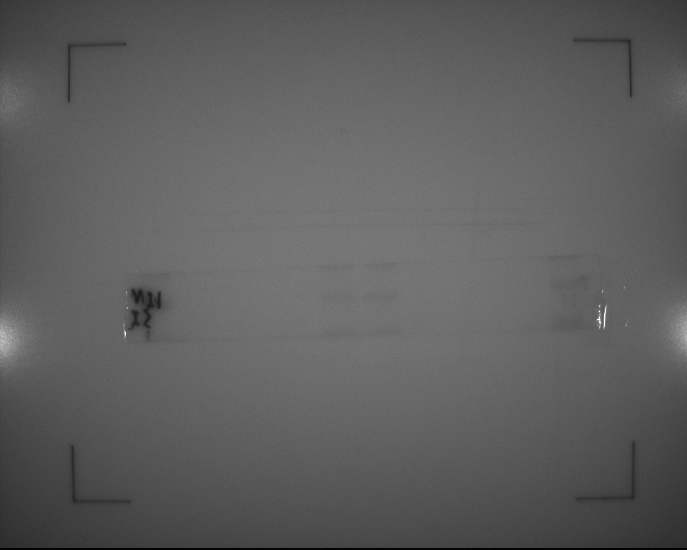

Supplement: Supplementary file 26 — Supplementary Material 26 [file 41598_2025_28877_MOESM26_ESM.tif]

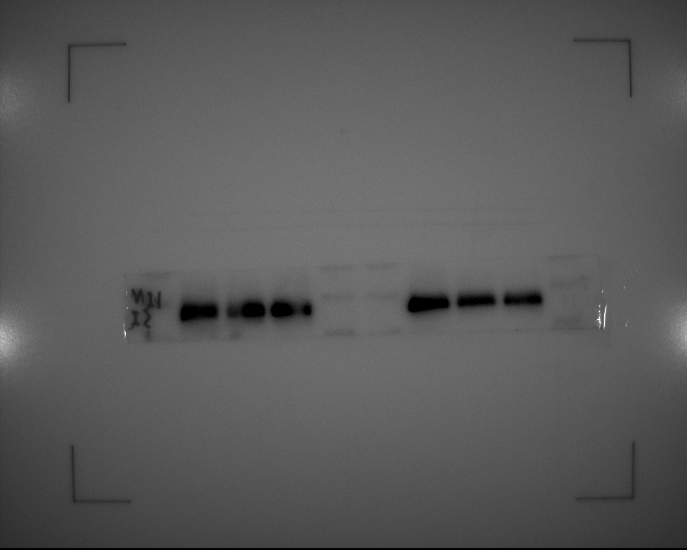

Supplement: Supplementary file 27 — Supplementary Material 27 [file 41598_2025_28877_MOESM27_ESM.tif]

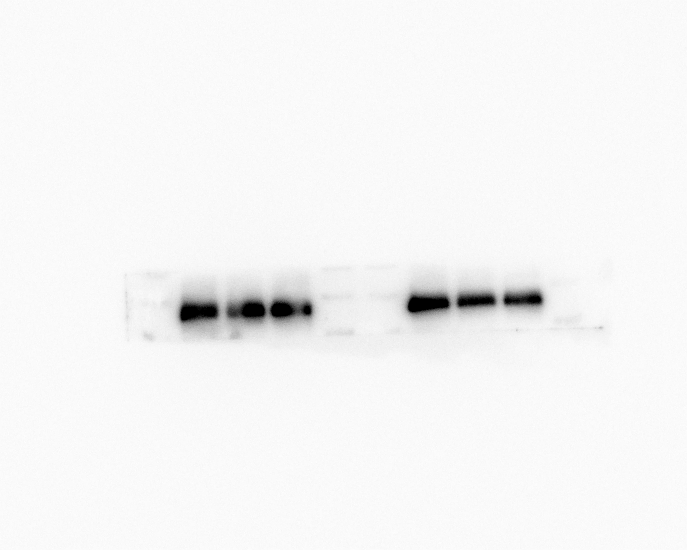

Supplement: Supplementary file 28 — Supplementary Material 28 [file 41598_2025_28877_MOESM28_ESM.tif]

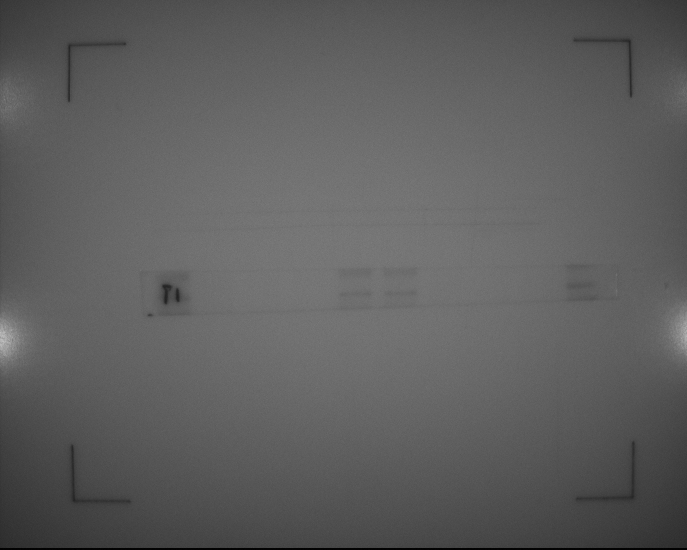

Supplement: Supplementary file 29 — Supplementary Material 29 [file 41598_2025_28877_MOESM29_ESM.tif]

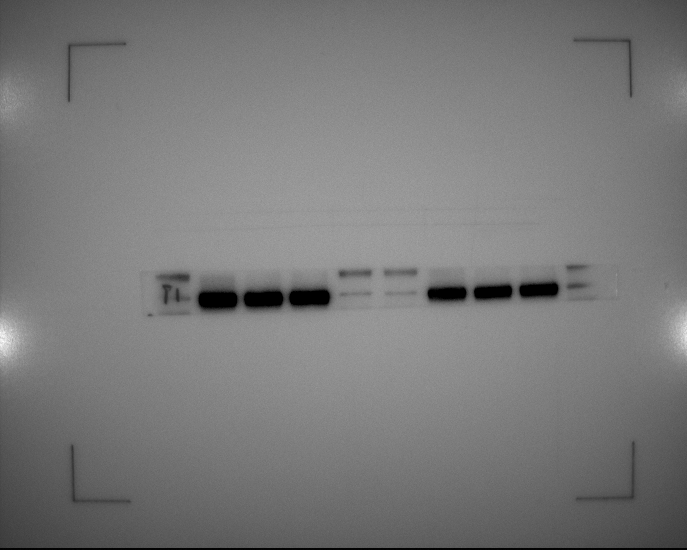

Supplement: Supplementary file 30 — Supplementary Material 30 [file 41598_2025_28877_MOESM30_ESM.tif]

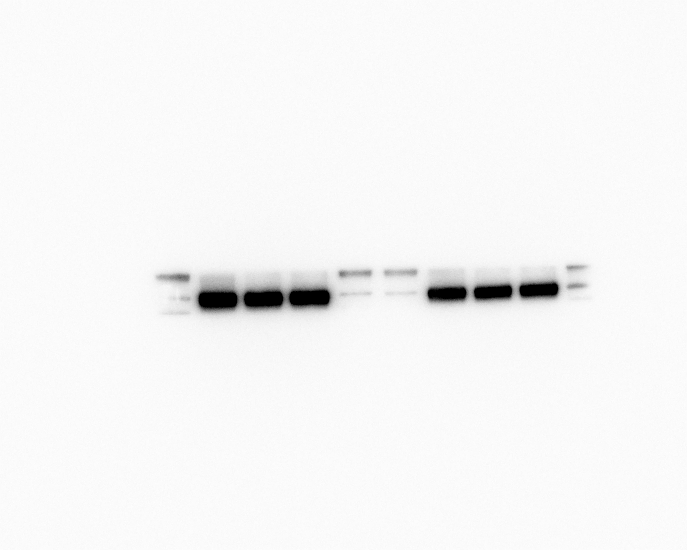

Supplement: Supplementary file 31 — Supplementary Material 31 [file 41598_2025_28877_MOESM31_ESM.tif]
